# Reproductive outcomes following recurrent first-trimester miscarriage: a retrospective cohort study

**DOI:** 10.1093/hropen/hoac045

**Published:** 2022-10-11

**Authors:** L A Linehan, I San Lazaro Campillo, M Hennessy, C Flannery, K O’Donoghue

**Affiliations:** INFANT Research Centre, University College Cork, Cork, Ireland; Pregnancy Loss Research Group, Department of Obstetrics & Gynaecology, University College Cork, Cork, Ireland; Pregnancy Loss Research Group, Department of Obstetrics & Gynaecology, University College Cork, Cork, Ireland; Pregnancy Loss Research Group, Department of Obstetrics & Gynaecology, University College Cork, Cork, Ireland; Pregnancy Loss Research Group, Department of Obstetrics & Gynaecology, University College Cork, Cork, Ireland; INFANT Research Centre, University College Cork, Cork, Ireland; Pregnancy Loss Research Group, Department of Obstetrics & Gynaecology, University College Cork, Cork, Ireland

**Keywords:** recurrent miscarriage, miscarriage, pregnancy, infertility, aneuploidy, counselling

## Abstract

**STUDY QUESTION:**

What are the subsequent reproductive outcomes (livebirths, miscarriages or other adverse pregnancy outcomes or no further pregnancy) of women with recurrent miscarriage (RM) attending a dedicated clinic?

**SUMMARY ANSWER:**

Of women with RM, 77% had a subsequent pregnancy, and among these pregnancies, the livebirth rate was 63%.

**WHAT IS KNOWN ALREADY:**

RM affects ∼1–3% of women of reproductive age. RM has known associations with advanced maternal age, obesity, diabetes, inherited thrombophilias, thyroid dysfunction, endometriosis and parental balanced translocations. However, ∼ 50% of women or couples will be left without an explanation for their pregnancy loss, even after completing investigations. RM is also associated with secondary infertility and adverse pregnancy outcomes including preterm birth and perinatal death.

**STUDY DESIGN, SIZE, DURATION:**

We undertook a retrospective cohort study to identify subsequent pregnancy outcomes in women with RM, defined as three consecutive first-trimester miscarriages. Women attending the RM clinic at a tertiary university hospital in the Republic of Ireland over 12 years (2008–2020) with a confirmed diagnosis of primary or secondary first-trimester RM were eligible for inclusion. In total, 923 charts were identified for review against the eligibility criteria.

**PARTICIPANTS/MATERIALS, SETTING, METHODS:**

Women with non-consecutive first-trimester miscarriages or ectopic pregnancy were excluded. Epidemiological and clinical information regarding medical history, investigation and management was gathered from paper and electronic medical records. Data were analysed using SPSS (Version 27). Associations between maternal characteristics and outcomes were explored using the χ^2^ test, with significance set at *P* < 0.05. Multinomial regression analysis was performed using a stepwise approach.

**MAIN RESULTS AND THE ROLE OF CHANCE:**

There were 748 women who were included; 332 (44%) had primary RM and 416 (56%) had secondary RM. The median age was 36 years (range 19–47). Foetal aneuploidy was the most common investigative finding (15%; n = 111/748); 60% had unexplained RM. In addition to supportive care, most women were prescribed aspirin (96%) and folic acid (75%). Of the 748 women, 573 had a subsequent pregnancy (77%) and 359 (48% of all women; 63% of pregnancies) had a livebirth, while 208 had a further pregnancy loss (28% of all women; 36% of pregnancies) and 6 were still pregnant at the end of the study. Women aged 35–39 years were more likely to have a livebirth than no further pregnancy (relative risk ratio (RRR): 2.29 (95% CI: 1.51–5.30)). Women aged 30–34 years were more likely to have a livebirth (RRR: 3.74 (95% CI: 1.80–7.79)) or a miscarriage (RRR: 2.32 (95% CI: 1.07–4.96)) than no further pregnancy. Smokers were less likely to have a livebirth (RRR: 0.37 (95% CI: 0.20–0.69)) or a miscarriage (RRR: 0.45 (95% CI: 0.22–0.90)) than no further pregnancy. Couples with an abnormal parental karyotype were less likely to have a miscarriage than no further pregnancy (RRR: 0.09 (95% CI: 0.01–0.79)). Including successive pregnancies conceived over the study period, the overall livebirth rate was 63% (n = 466/742), but this was reduced to 44% in women aged ≥40 years and 54% in women with infertility.

**LIMITATIONS, REASONS FOR CAUTION:**

This work covers 13 years; however, those included in the later years have a shorter follow-up time. Although electronic health records have improved data availability, data collection in this cohort remains hampered by the absence of a formal booking visit for women presenting with miscarriage and a national miscarriage database or register.

**WIDER IMPLICATIONS OF THE FINDINGS:**

Our findings are largely reassuring as most women with RM and hoping to conceive achieved a livebirth. In addition to older age, smoking and parental balanced translocations were associated with a reduced likelihood of further pregnancy. No investigation or treatment was associated with pregnancy outcome, reiterating the importance of the supportive aspects of care for women and their partners after RM and counselling regarding individual risk factors. This contributes to the limited international data on the investigative findings and treatment of women with RM. The high rate of prescribed medications merits greater scrutiny, in conjunction with other pregnancy outcomes, and reiterates the need for a national guideline on RM.

**STUDY FUNDING/COMPETING INTEREST(S):**

L.A.L. is a PhD scholar funded through the Pregnancy Loss Research Group, Department of Obstetrics and Gynaecology, University College Cork. M.H. and C.F. are Postdoctoral Researchers on a project funded by the Health Research Board Ireland [ILP-HSR-2019-011] and led by K.O.D., titled: ‘Study of the impact of dedicated recurrent miscarriage clinics in the Republic of Ireland’. The funders had no role in study design, data collection and analysis, decision to publish or preparation of the manuscript. The authors have no conflicts of interests to declare.

**TRIAL REGISTRATION NUMBER:**

N/A.

WHAT DOES THIS MEAN FOR PATIENTS?

We studied women who had experienced three miscarriages to see if they had a livebirth, another pregnancy loss or no further pregnancies. We investigated whether features such as medical history, investigations and treatments for recurrent miscarriage were linked to their pregnancy outcome. We found that 77% of women conceived; of these 63% had a livebirth and 36% had a further pregnancy loss. Maternal age, smoking and parental genetic conditions were linked to whether or not a woman conceived and had a baby.

## Introduction

A miscarriage is defined as the spontaneous demise of a pregnancy before the foetus reaches viability (24 weeks gestation in the UK and Ireland) (RCOG, 2011). Miscarriage is the most common pregnancy complication, with the most recent data estimating 23 million miscarriages worldwide per year (Quenby *et al.*, 2021).

Recurrent pregnancy loss (RPL) must be distinguished from recurrent miscarriage (RM); RPL is defined as any two non-consecutive losses before viability (Practice Committee of the ASRM, 2012; ESHRE Early Pregnancy Guideline Development Group, 2017), whereas RM has been defined as three consecutive first-trimester miscarriages (RCOG, 2011; ESHRE Early Pregnancy Guideline Development Group, 2017; Toth *et al.*, 2018). The population prevalence is ∼1.9% for women with two miscarriages and 0.7% for three or more (Quenby *et al.*, 2021). Risk factors for RM include maternal age (>35 years), paternal age, maternal BMI, number of previous miscarriages, smoking, Black ethnicity, alcohol and stress, of which the strongest association is with maternal age (Quenby *et al.*, 2021). RM is associated with subsequent adverse pregnancy outcomes, including antepartum haemorrhage, gestational diabetes, preterm birth, small for gestational age and perinatal death (Chen *et al.*, 2018; Wu *et al.*, 2022). The risk of preterm birth, in particular, rises with each successive miscarriage (Quenby *et al.*, 2021). Moreover, RM is associated with significant psychological distress extending to post-traumatic stress disorders, anxiety, depression and suicidality (Farren *et al.*, 2020). Therefore, supportive care and reassurance scans are of significant psychological benefit to couples with RM (Musters *et al.*, 2013).

International guidelines recommend that for women with RM, a detailed medical history should direct investigations and treatments (RCOG, 2011; ESHRE Early Pregnancy Guideline Development Group, 2017). Associated medical conditions include antiphospholipid syndrome (APLS), inherited thrombophilias, thyroid disease, uterine anomalies and parental chromosomal rearrangement, and are the focus of investigations alongside foetal chromosomal analysis for explanatory purposes (Coomarasamy *et al.*, 2021). After investigations, ∼38–60% of women will have unexplained RM (Clifford *et al.*, 1994; Jaslow *et al.*, 2010; Fawzy *et al.*, 2016; Ali *et al.*, 2020). There is no high-quality evidence for any treatments in miscarriage prevention; however, there is some evidence for the use of progesterone in women with a previous miscarriage with bleeding in a subsequent pregnancy, thyroxine in sub-clinical hypothyroidism and low-molecular-weight heparin (LMWH) and aspirin in women with APLS (Coomarasamy *et al.*, 2021). It is recommended that women are counselled about investigative findings and treatments in a specialized RM clinic setting, where additional supportive management can also be instigated (RCOG, 2011; ESHRE Early Pregnancy Guideline Development Group, 2017).

Inconsistent recommendations and definitions have resulted in a variance in when and how women with RM are investigated and treated (Manning *et al.*, 2020). A UK survey on the care of women with RM demonstrated that just a third of respondents had attended a dedicated RM clinic and that investigation and treatment deviated significantly from RCOG guidelines, often at the women’s request (Manning *et al.*, 2020). A Lancet Series on Miscarriage also noted the significant economic burden and psychological impact of RM on women and couples (Quenby *et al.*, 2021). Their suggested graded approach advocates for earlier access to psychological supports, reassurance scans and preliminary investigations (Coomarasamy *et al.*, 2021). This is reflected in drafts of updated guidelines on RM which suggest investigation after two non-consecutive miscarriages (Regan *et al.*, 2021; ESHRE Early Pregnancy Guideline Development Group, 2022).

In the Republic of Ireland, 19 maternity units provide obstetric care to women and their families. The structure of RM care in these individual units and the specifics of care offered are not clear. Nationally, there is no data collection pertaining to miscarriage or RM; thus, incident rates are unknown, as are the maternal characteristics and subsequent pregnancy outcomes, which is a sizeable gap in the literature. An Irish cohort study demonstrated that women with RM were more likely to have a BMI ≥30, to have had assisted conception or a previous perinatal death, and were more likely to have a further preterm birth or perinatal death, compared to women with no RM history (Field and Murphy, 2015). While this contributes to the limited international data on pregnancy outcomes following RM, it is unclear what percentage of these women attended a RM clinic and what investigations, treatments or supports were provided (Field and Murphy, 2015).

The pregnancy loss service in Cork University Maternity Hospital (CUMH) was established in 2008 to provide specialized medical care and support to bereaved women and their families (which has always included women with primary RM or secondary RM, i.e. RM after a previous viable birth), who are seen within a specialized RM clinic. This study aimed to address the following research question: what were the subsequent reproductive outcomes for women who attended this RM clinic with three or more consecutive first-trimester miscarriages? More specifically, what were the different maternal characteristics, investigations and treatments associated with a livebirth, a further pregnancy loss or no further pregnancy after RM, and what was the overall livebirth rate in the cohort during the study period?

## Materials and methods

### Study design

A retrospective cohort study was conducted to examine subsequent reproductive outcomes for women with three or more consecutive first-trimester miscarriages. This study is reported in accordance with the Strengthening the Reporting of Observational Studies in Epidemiology (STROBE) Statement (Von Elm *et al.*, 2007).

Women with a confirmed history of primary or secondary first-trimester RM attending the RM clinic at CUMH from 1 January 2008 to 31 December 2020 were included. The cohort was followed until 28 February 2022 to identify any subsequent pregnancies. This study was conducted at CUMH, a large tertiary university hospital in the Republic of Ireland with ∼8000 births per year. The RM clinic is a consultant-led clinic, with ongoing supportive care provided by Clinical Midwife Specialists (CMS) in Bereavement and Loss. Approximately 70 women/couples a year are seen in the clinic to discuss results of RM investigations and potential treatments. All women who attended the RM clinic met with a CMS at their appointment and had supportive follow-up as required. Additionally, women were facilitated with an early ultrasound scan in a subsequent pregnancy and recommended to attend the consultant-led perinatal medicine antenatal clinic.

### Ethical approval

Ethical approval for this study was obtained from the Clinical Research Ethics Committee of the Cork Teaching hospitals (ECM 6 (m) 6 December 2016 and ECM 3 (z) 10 January 2017).

### Study population

Women were identified from the clinical database of the RM clinic and inpatient registers. Paper and electronic charts, clinic letters, radiology records, electronic hospital laboratory systems and the Profile Information Management System (PIMS) were reviewed to confirm inclusion eligibility and gather information on primary and secondary outcome measures. Women who had non-consecutive first-trimester miscarriages or whose three consecutive losses included an ectopic pregnancy, second-trimester miscarriage (defined as a pregnancy loss at 13–23 + 6 weeks gestation), stillbirth (defined as loss after 24 weeks gestation or of a foetus weighing ≥500 g) or termination of pregnancy were excluded as per international definitions of RM (RCOG, 2011; ESHRE Early Pregnancy Guideline Development Group, 2017). All women with secondary RM were confirmed as having had at least three consecutive miscarriages, but fourth and subsequent miscarriages were not necessarily consecutive. Women who were previously seen for preliminary investigations after two miscarriages and who returned for additional investigations and treatment after a third consecutive miscarriage were included. A minority of women who attended a different consultant within the hospital group for RM care were included if clinical correspondence outlining their history, investigations and treatment was available and if these were in keeping with the RM clinic protocols. Women who attended the RM Clinic for investigations and treatment but later received antenatal care or gave birth in another unit were excluded.

### Outcome measure

The primary outcome measure was the reproductive outcome for women following at least three consecutive miscarriages, either a livebirth, a further pregnancy loss (which included first or second-trimester miscarriage, stillbirth, ectopic pregnancy or termination of pregnancy) or no subsequent pregnancy. If women who had experienced a further pregnancy loss in their first pregnancy after RM had any additional successful pregnancy in the study period, this was also recorded to determine the overall livebirth rate.

### Covariates

#### Sample characteristics

Maternal characteristics included age (≤29, 30–34 years, 35–39 years, ≥40 years), primary RM versus secondary RM, smoking (current, non-smokers, previous smokers, unknown smoking status) and BMI (<25, ≥25, not documented). If data on medical history, gynaecological conditions, gynaecological procedures, fertility history, assisted reproductive therapy history and male partner history were available; this was recorded and coded as binary data (present/absent). Maternal age was recorded as age at attendance at RM clinic, as were other characteristics, if available.

#### Investigations and treatments

Routine investigations within the RM clinic and typically recommended pharmacological treatments with the standard dosages are presented in Table I. The types of investigations were recorded, along with the results (normal versus abnormal). For treatment, dosing and timings varied for some patients (∼3%) (e.g. 150 mg aspirin, aspirin from 6 weeks gestation only, progesterone 400 mcg twice daily or therapeutic doses of LMWH). Therefore, this analysis was restricted to whether an individual drug was prescribed, and not its specific dosage or timing.

**Table I:** Data collected for investigations and treatments.

Investigations	Notes
**Thrombophilia screen**	Factor V Leiden screen
	Anti-cardiolipin antibodies
	Lupus anticoagulant
	Protein S
	Protein C
	Anti-thrombin III
**Prothrombin gene**	Previously part of thrombophilia screen but due to a change in laboratory policy during the study period was only performed when a FVL screen was positive
**Autoantibody screen**	Anti-nuclear antibodies
	Extractable nuclear antigen
	Anti-neutrophil cytoplasmic antibody, rheumatoid factor
	Others, as clinically indicated
**Thyroid function tests**	Thyroid-stimulating hormone (raised if <2.5 mIU/l) Thyroid antibodies (present/absent)
**HbA1c**	≥39 mmol/mol
**Pelvic ultrasound**	Positive ultrasound findings were included as a positive investigative finding unless there was adequate clinical information to regard the finding as insignificant, e.g. ovarian cysts <5 cm
**Foetal karyotyping**	Foetal karyotyping was performed if pregnancy tissue was available at the time of a third or subsequent miscarriage
**Parental karyotyping**	Performed on both partners together if possible
**Treatments:**	
**Folic acid**	5 mg
**Aspirin**	75 mg
**Progesterone**	400 µg vaginally once daily
**Low molecular weight Heparin**	Prophylactic dose once daily subcutaneously (typically 4500 iu tinzaparin)
**Prednisolone**	20 mg twice daily
**Metformin**	500 mg twice daily
**Hydroxychloroquine**	200 mg once daily

In keeping with clinical practice and international guidelines, we focused on Factor V Leiden (FVL) Prothrombin (PT) gene, Lupus anticoagulant and anti-cardiolipin antibodies (ACLA) in the thrombophilia screen (Coomarasamy *et al.*, 2021), and the remaining thrombophilias were grouped as one covariate ‘other thrombophilia’. Diagnosis of antiphospholipid was made in women with abnormal antiphospholipid antibodies (lupus anticoagulant, ACLA and anti-B2 glycoprotein-I antibodies) associated with a history of adverse pregnancy outcome and vascular thrombosis. The diagnosis was confirmed by two positive tests 3 months apart as per international guidelines (Keeling *et al.*, 2012).

A finding of autoantibodies alone or a weak lupus anticoagulant were grouped as ‘tests of uncertain significance’ when considering abnormal investigations.

### Data collection

Data from 2008 to 2016 were collected by four clinical staff and managed by a researcher (I.S.L.C.). These data were then verified and merged with the 2017–2020 data, which the primary author collected. Data collected from clinical notes or correspondence were confirmed by checking laboratory and radiology systems and inpatient records. Data were stored securely as per hospital and university data protection guidelines.

Collected data were entered into a Microsoft Excel file. The data were cleaned, and relevant variables were entered into SPSS, version 27, for analysis. Associations between participant characteristics and subsequent reproductive outcomes were explored using the χ^2^ test for categorical variables with significance defined as *P* < 0.05. Similar analysis was undertaken to investigate associations between participant characteristics and subsequent pregnancy outcomes, i.e. livebirth or a further pregnancy loss.

Multinomial logistic regression was conducted using a stepwise approach. Model 1 included maternal characteristics and Model 2 added the RM investigations. Only variables that were statistically significant in the unadjusted regression model were included. Estimated coefficients are reported as relative risk ratios (RRRs) with 95% CI using women who had no subsequent pregnancy as the reference category. This is because the exponentiated coefficient in multinomial logistic regression is the ratio of two relative risks and should not be interpreted as an odds ratio.

## Results

**Figure 1.**
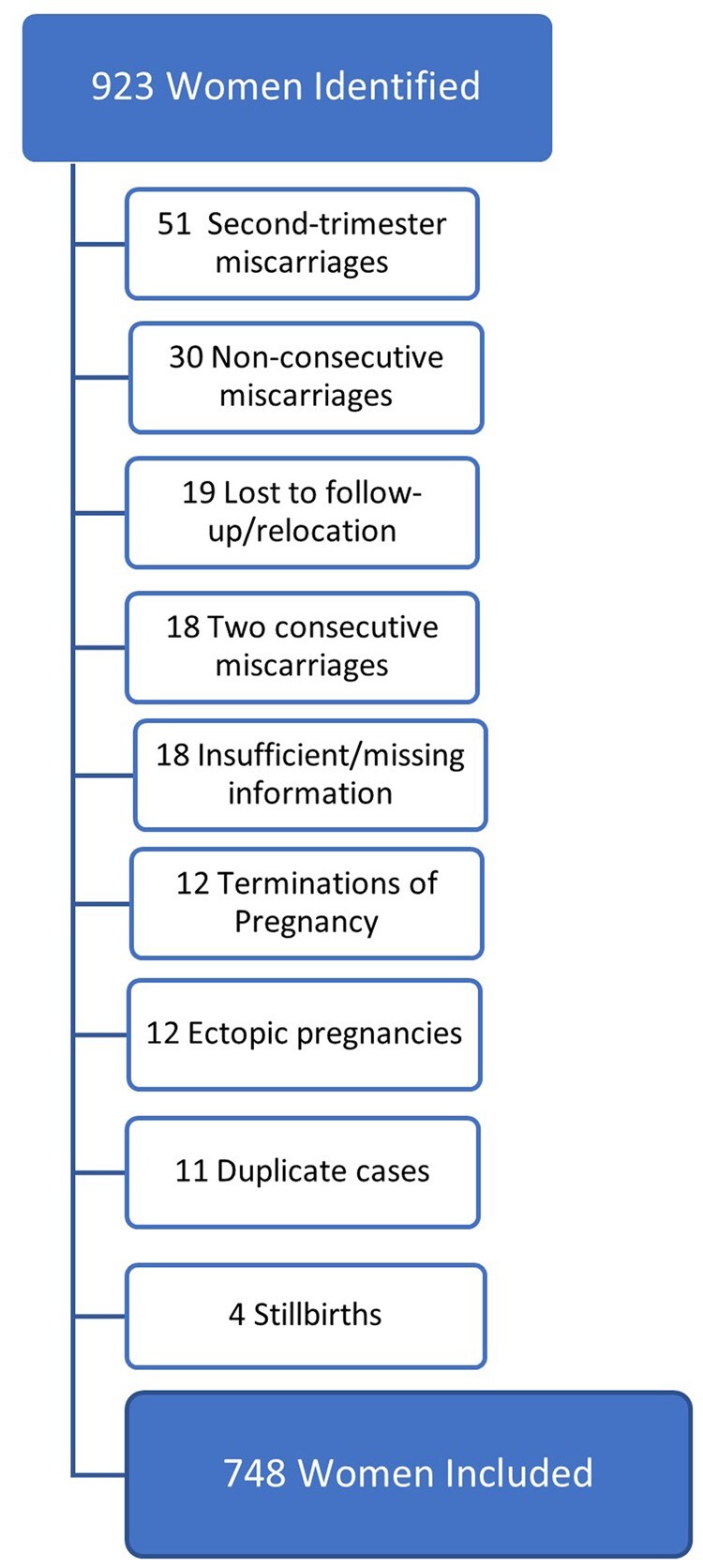
**Women excluded from the study of reproductive outcomes following recurrent miscarriage**.

A review of the RM clinic database and inpatient registers between 2008 and 2020 identified 923 charts for analysis; of these, 748 women with RM were eligible for inclusion in the study. Exclusions are presented in Fig. 1. The median follow-up time was 7 years 1 month (range 1 year 2 months to 14 years).

### Maternal characteristics

The median age in this cohort was 36 years (range: 19–47, standard deviation: 5.06). Most women experienced a secondary RM (55.6%, n = 416), were non-smokers (80.4%, n = 403 of 501) and had no documented medical history (73.5%, n = 535 of 728) (see Table II). Furthermore, 81% of women had no documented history of infertility (n = 606).

**Table II:** Maternal characteristics at the time of recurrent miscarriage clinic attendance.

Demographics (n = 748)	N	Frequency	Percentage (%)
**Age**	748		
<29		89	11.9
30–34		182	24.3
35–39		283	37.8
>40		194	25.9
**Recurrent miscarriage**	748		
Primary		332	44.4
Secondary		416	55.6
**Previous adverse pregnancy outcomes**	748		
Stillbirth		3	0.4
Preterm birth		18	2.4
**Previous livebirths**	748		
0		333^*^	44.5
1		264	35.2
2		110	14.7
3		29	3.9
4+		12	1.6
**No of miscarriages**	748		
3		483	64.6
4		166	22.2
5		57	7.6
6		25	3.3
7		11	1.5
8		1	0.1
9		2	0.3
10		2	0.3
12		1	0.1
**Smoking status**	501		
Current smoker		85	17.0
Non-smoker		403	80.4
Previous smoker		13	2.6
**Body mass index**	287		
<25		218	76.0
>25		61	21.3
>40		8	2.8
**Documented medical history**	728		
No medical history		535	73.5
Medical history		193	26.5
**Recorded medical conditions**	748		
Hypothyroidism		48	6.4
Autoimmune disorder		24	3.2
Mental health disorder		22	2.9
**Documented gynaecological history**	728		
Gynaecological history		50	6.9
No gynaecological history		678	93.1
**Gynaecological conditions**	39		
Polycystic ovary syndrome (PCOS)		18	46.2
Endometriosis		12	30.8
Fibroids		9	23.1
**Documented gynae-surgical procedure(s)**	727		
Gynaecological procedure(s)		142	19.5
No gynaecological procedure(s)		585	80.5
**Gynaecological procedures**	108		
Caesarean section		43	39.8
Surgical management of miscarriage		34	31.5
Large loop excision of transitional zone		16	14.8
Diagnostic laparoscopy ± dye		15	13.9
**Documented fertility history**	748		
Fertility history		142	19.0
No fertility history		606	81.0
**Fertility type**	142		
RM as primary reason for investigations		46	32.4
Unexplained primary infertility		19	13.4
Prolonged time to conception		15	10.6
Prior IVF (reason undocumented)		15	10.6
Anatomical cause		10	7.0
Male factor		8	5.6
Unexplained secondary infertility		7	4.9
Polycystic ovary syndrome (PCOS)		7	4.9
Endometriosis		6	4.2
Advanced maternal age		5	3.5
Balanced translocation		3	2.1
Premature ovarian failure		1	0.7
**ART type**	66		
IVF		25	37.9
Ovulation induction		12	18.2
IUI		11	16.7
Oocyte donation		9	13.6
ICSI		3	4.5
Preimplantation genetic testing (PGT)		2	3.0
IVF—reasons undocumented		4	6.0

* Recorded as having had no previous live birth (stillbirth in first pregnancy: n = 1).

Previous delivery information was available for 201 women with secondary RM (48%; 201/416); 142 (71%) had a prior vaginal birth, with the remainder having at least one caesarean section (n = 59, 29%). Of 265 women with four or more miscarriages, 76% had at least one previous livebirth (n = 202/265). Of the 42 women with six or more miscarriages, 26% of women had no previous livebirth (11/42).

### Investigations performed

Almost all women had the RM clinic standard investigations performed (727/748; 97%). An overview of these investigations and prescribed treatments is provided in Table III.

**Table III:** Details of standard investigations results and prescribed treatments.

Investigation	N = 748 (%)	Frequency	Percentage
**Prothrombin gene**	344 (46)		
Mutation present		7	2.0
Mutation absent		337	98.0
**Factor V Leiden**	737 (98.5)		
Mutation present		35	4.7
Mutation absent		702	95.3
**Anti-cardiolipin antibodies**	740 (99)		
Present		10	1.4
Absent		730	98.6
Re-test positive and APLS diagnosed		4	5.4
**Other thrombophilia**	740 (99)		
Weakly+ lupus anticoagulant		5	0.7
Protein S deficiency		1	0.1
**All autoantibodies**	743 (99)		
One or more present		94	12.7
No antibodies		655	87.3
Anti-nuclear antibodies present		89	12.0
**HbA1c**	742 (99)		
Elevated		7	1.0
Not elevated		735	99.0
**Thyroid function tests**	742 (99)		
Normal		683	92.0
Abnormal		59	8.0
**Previous foetal karyotype**	141 (19)		
Euploid		30	21.3
Aneuploid		111	78.7
**Most common aneuploidies**	111		
Trisomy 16		17	15.3
Trisomy 21		14	12.6
Trisomy 22		14	12.6
Trimsomy15		10	9.0
Triploidy		10	9.0
Trisomy 13		8	7.2
45XO		6	5.4
**Parental karyotype**	697 (93)		
Balanced translocation present^*^		28	4.0
Normal karyotype		669	96.0
**Pelvic ultrasound**	748 (100)		
Finding on US		46	6.1
No finding		702	93.9
**Most common US findings**	46		
Polycystic ovaries		13	28.3
Uterine fibroids		11	23.9
Bicornuate uterus		10	21.7
**Medical therapies initiated**			
**Aspirin**	728 (97)	696	96
**Folic Acid 5 mg**	728 (97)	548	75
**Progesterone**	728 (97)	389	52
**Low-molecular-weight heparin**	727 (97)	175	24
**Prednisolone**	727 (97)	28	4
**Metformin**	727 (97)	12	2
**Hydroxychloroquine**	727 (97)	7	1

* Nineteen maternal and 9 paternal balanced translocations.

APLS, anti-phospholipid syndrome; US, ultrasound.

Overall, 297 women had at least one investigative finding (297/748; 39.7%); 53 women had two or more positive results and six women had three. The most common positive finding was an abnormal foetal karyotype (n = 111), followed by a positive anti-nuclear antibodies (ANA) result (n = 89), abnormal thyroid function tests (n = 59), a finding of FVL (n = 35) or a balanced translocation in either parent (n = 28). Eight women had a sole positive finding of a test of uncertain significance.

### Treatments

Prescribed medical therapies are also outlined in Table III. In addition to supportive care and medications, cervical surveillance was recommended in a subsequent pregnancy for seven women and three women with a history of preterm birth were advised to have a cervical cerclage.

### Outcomes

**Figure 2.**
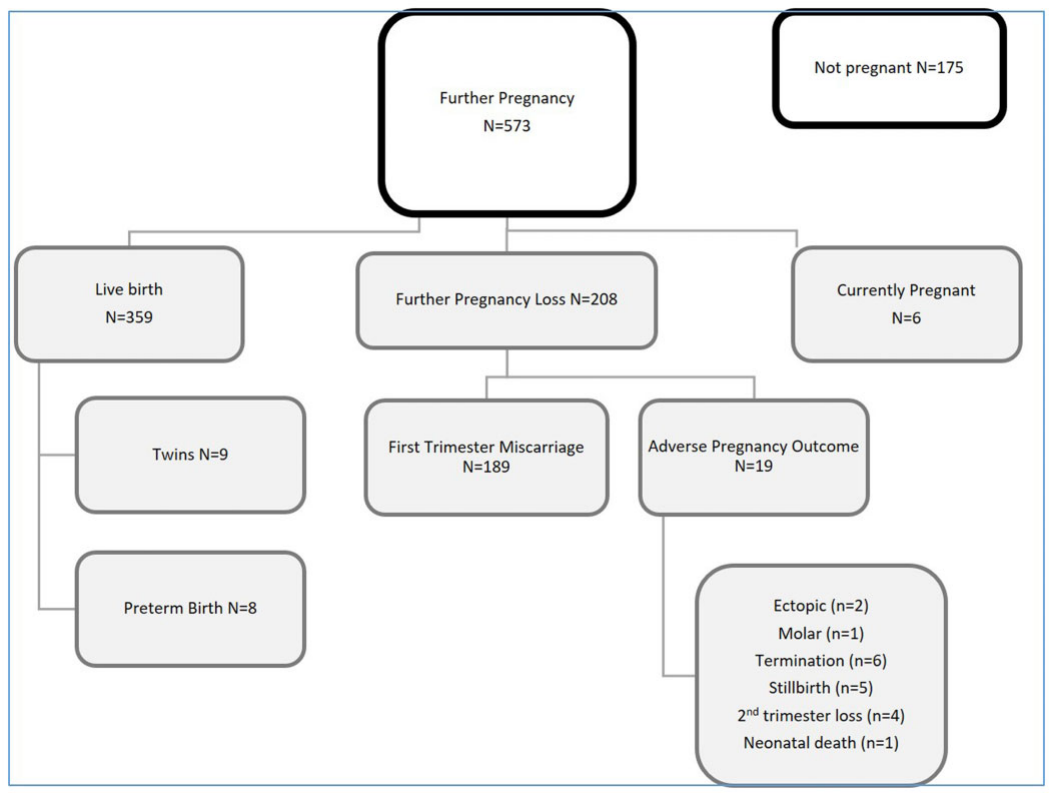
**Reproductive outcomes for women in their first pregnancy after recurrent miscarriage**.

There were 573 women (77%) who had a subsequent pregnancy following at least three consecutive miscarriages; the majority of whom conceived within 1 year of attending the RM clinic (441/573; 77%). Of the 573 women, 93% (531) had at least one early pregnancy ultrasound scan before their routine booking ultrasound at 12 weeks. The livebirth rate overall was 48% (359/748) and 63% among the women who became pregnant (359/573), while there were six additional pregnancies still ongoing at the end of the follow-up period; other pregnancy outcomes are shown in Fig. 2.

Including successive pregnancies over the study period, the cumulative livebirth rate among those who had one or more subsequent pregnancies was 81% (466/573) and 63% (466/742) among all of the women with RM (after excluding the six women with ongoing pregnancies). When examined according to age, the cumulative livebirth rate for the 742 women with RM was 67% in women aged under 30 (n = 59/88), 73% in women aged 30–34 (n = 131/180), 68% in those aged 35–39 years (n = 191/281) and 44% in women aged over 40 (n = 85/193) (*P* < 0.001). The cumulative livebirth rate for women with any history of infertility was 54% (n = 75/140), compared to 65% for those with no infertility history (n = 391/602) (*P* = 0.019).

### Maternal characteristics versus reproductive outcome

Maternal characteristics were examined compared to reproductive outcomes (livebirth, pregnancy loss or no further pregnancy), and are shown in Table IV. Based on the chi-square test, age was associated with reproductive outcome (*P* < 0.01), as was current smoking (*P* = 0.011), a history of an abnormal karyotype in either partner (*P* = 0.014) and treatment with progesterone (*P* = 0.007). Specifically, women ≥40 had a lower livebirth rate (32%), higher miscarriage rate (34%) and higher rate of no further pregnancy (34%) compared to younger women in the cohort. Smokers and couples with balanced translocations had higher rates of no further pregnancy and women prescribed progesterone had a higher rate of pregnancy loss.

**Table IV:** Maternal characteristics versus reproductive outcome.

Maternal characteristic (n, %)	Livebirth n (%)	Pregnancy Loss	No further pregnancy	*P*-value
	(N = 359)	(N = 208)	(n = 175)	
**Maternal age**				
≤29	45 (51.1)	21 (23.9)	22 (25)	
30–34	102 (56.7)	54 (30)	24 (13.3)	
35–39	151 (53.7)	67 (23.8)	63 (22.4)	
≥40	61 (31.6)	66 (34.2)	66 (34.2)	<0.001
**Previous livebirth**				
Yes	202 (49.0)	111 (26.9)	99 (24.0)	
No	157 (47.6)	97 (29.4)	76 (23.0)	0.759
**Medical history^a^**				
Yes	95 (44.2)	60 (27.9)	60 (27.9)	
No	259 (50.8)	144 (28.2)	107 (21.0)	0.105
**Gynaecological condition^b^**				
Yes	18 (37.5)	14 (29.2)	16 (33.3)	
No	333 (49.4)	188 (27.9)	153 (22.7)	0.174
**Gynaecological procedure^c^**				
Yes	64 (45.7)	36 (25.7)	40 (28.6)	
No	287 (49.4)	166 (28.6)	128 (22.0)	0.257
**Body mass index**				
Not documented	222 (48.3)	128 (27.8)	110 (23.9)	
<25	103 (48.1)	61 (28.5)	50 (23.4)	
>25	34 (50.0)	19 (27.9)	15 (22.1)	0.997
**Smoker^d^**				
Yes	35 (41.8)	20 (23.5)	29 (34.5)	
No	212 (51.5)	119 (28.9)	81 (19.7)	0.011
**Fertility history**				
Yes	58 (41.4)	40 (28.6)	42 (30.0)	
No	301 (50.0)	168 (27.9)	133 (22.1)	0.093
**ART history**				
Yes	31 (47.0)	22 (33.3)	13 (19.7)	
No	328 (48.5)	186 (27.5)	162 (24.0)	0.543
**Prothrombin gene**				
Not performed	189 (47.5)	109 (27.4)	100 (25.1)	
Mutation present	3 (42.9)	4 (57.1)	0 (0.0)	
Mutation absent	167 (49.6)	95 (28.2)	75 (22.3)	0.321
**Factor V Leiden^e^**				
Mutation present	20 (57.1)	6 (17.1)	9 (25.7)	
Mutation absent	335 (48.1)	199 (28.6)	163 (23.4)	0.334
**Anti-cardiolipin antibodies^f^**				
Present	6 (54.5)	4 (36.4)	1 (9.1)	
Absent	351 (48.5)	200 (27.7)	172 (23.8)	0.504
**All autoantibodies^g^**				
One or more present	48 (51.1)	24 (25.5)	22 (23.4)	
No antibodies	309 (48.0)	183 (28.5)	151 (23.5)	0.817
**HbA1c^g^**				
Elevated	2 (28.6)	2(28.6)	3 (42.9)	
Not elevated	355 (48.6)	205 (28.1)	170 (23.3)	0.426
**Thyroid function tests^h^**				
Normal	329 (48.5)	196 (28.9)	153 (22.6)	
Abnormal	28 (48.3)	11 (19)	19 (32.8)	0.119
**Previous foetal karyotype (n = 129)**				
Aneuploid	54 (49.1)	28 (25.5)	28 (25.5)	
Euploid	8 (27.6)	10 (34.5)	11 (37.9)	0.114
**Parental karyotype^i^**				
Balanced translocation present	13 (46.4)	3 (10.7)	12 (42.9)	
Normal karyotype	326 (49.2)	193 (29.1)	144 (21.7)	0.014
**Pelvic ultrasound**				
Finding on US	21 (46.7)	15 (33.3)	9 (20.0)	
No finding	338 (48.5)	193 (27.7)	166 (23.8)	0.68
**Any positive investigation finding**				
Yes	155 (48.7)	85 (26.7)	78 (24.5)	
No	204 (48.1)	123 (29.0)	97 (22.9)	0.755
Prescribed aspirin^*^				
Yes	336 (48.7)	196 (28.4)	18 (22.9)	
No	14 (46.7)	9 (30)	7 (23.3)	0.974
Prescribed folic acid 5 mg^*^				
Yes	257 (47.3)	161 (29.7)	125 (23.0)	
No	95 (53.1)	44 (24.6)	40 (22.3)	0.340
Prescribed progesterone^*^				
Yes	175 (45.5)	129 (33.5)	81 (21.0)	
No	177 (52.5)	77 (22.8)	83 (24.6)	0.007
**Prescribed LMWH^§^**				
Yes	76 (44.4)	52 (30.4)	43 (25.1)	
No	276 (50.2)	153 (27.8)	121 (22)	0.416
**Prescribed prednisolone^§^**				
Yes	12 (44.4)	11 (40.7)	4 (14.8)	
No	339 (48.9)	194 (28.0)	160 (23.1)	0.306
**Prescribed metformin^§^**				
Yes	5 (41.7)	3 (25.0)	4 (33.3)	
No	347 (48.9)	202 (28.5)	160 (22.6)	0.677
**Prescribed hydroxychloroquine^§^**				
Yes	3 (50.0)	3 (50.0)	0 (0.0)	
No	349 (48.8)	202 (28.3)	164 (22.9)	0.306

a Missing data (n = 17).

b Missing data (n = 20).

c Missing data (n = 21).

d Missing data (n = 244).

e Missing data (n = 10).

f Missing data (n = 8).

g Missing data (n = 5).

h Missing data (n = 6).

i Missing data (n = 41).

* n = 728.

§ n = 727.

LMWH, low-molecular-weight heparin; US, ultrasound.

To determine any differences between women who had a livebirth and those who had a further loss, a chi-square test was performed on maternal characteristics that were associated with reproductive outcomes as per Table IV (see Supplementary Table SI). Only age ≥40 was shown to be associated with having a further pregnancy loss. A previous livebirth was not associated with achieving a further pregnancy or a livebirth (Table IV and Supplementary Table SI). Binary regression analysis was not performed to compare maternal characteristics and pregnancy outcome as the data were too small in some covariate groups to be conclusive.

### Multinomial logistic regression analysis

In the unadjusted multinomial logistic regression, maternal age, any medical history, current smoking, any infertility history, abnormal thyroid function tests, parental balanced translocations and prescribed progesterone were linked to either livebirth or a further pregnancy loss (see Supplementary Table SII).

In the fully adjusted model, age, smoking status and the presence of a parental balanced translocation remained associated with pregnancy outcome (Table V). With respect to women aged over 40, women aged 35–39 were 2.3 times more likely to have a livebirth than no further pregnancy (RRR: 2.29 (95% CI: 1.51–5.30)), and women aged 30–34 were more likely to have a livebirth (RRR: 3.74 (95% CI: 1.80–7.79)) or a miscarriage (RRR: 2.31 (95% CI: 1.07–4.96)) than no further pregnancy. With respect to non-smokers, smokers were less likely to have a livebirth (RRR: 0.37 (95% CI: 0.20–0.69)) or a miscarriage (RRR: 0.45 (95% CI: 0.22–0.90)) than no further pregnancy. With respect to couples with normal parental karyotypes, couples with an abnormal parental karyotype were less likely to have a miscarriage than no further pregnancy (RRR: 0.09 (95% CI: 0.01–0.79)).

**Table V:** Adjusted multinomial regression analysis of maternal characteristics versus reproductive outcome.

*Variable*		Model 1^*^				Model 2^*^		
	Live birth^a^	*P*-value	Further pregnancy loss^a^	*P*-value	Live birth^a^	*P*-value	Further pregnancy loss^a^	*P*-value
	RRR (95% CI)		RRR (95% CI)		RRR (95% CI)		RRR (95% CI)	
**Maternal characteristics**								
**Age category**	1.95 (0.89–4.28)	0.096	0.61 (0.24–1.54)	0.294	2.03 (0.90–4.61)	0.089	0.73 (0.277–1.91)	0.517
<29 years								
30–34 years	3.99 (1.94–8.22)	<0.010	2.33 (1.11–4.91)	0.026	3.74 (1.80–7.79)	<0.01	2.31 (1.07–4.96)	0.033
35–39 years	2.54 (1.40–4.61)	0.010	1.15 (0.61–2.15)	0.669	2.29 (1.51–5.30)	0.001	1.33 (0.68–2.61)	0.402
≥40 years^b^	1		1		1		1	
**Medical history**								
Yes	0.675 (0.41–1.12)	0.127	0.86 (0.50–1.48)	0.581	0.71 (0.42–1.21)	0.210	0.98 (0.55–1.75)	0.946
No^b^	1		1		1		1	
**Fertility history**								
Yes	0.79 (0.45–1.40)	0.422	0.82 (0.44–1.52)	0.523	0.74 (0.41–1.34)	0.320	0.70 (0.370–1.34)	0.284
No^b^	1		1		1		1	
**Smoking**								
No^b^	1		1		1		1	
Current smokers	0.43 (0.24–0.79)	0.006	0.52 (0.26–1.01)	0.053	0.37 (0.20–0.69)	0.020	0.45 (0.22–0.90)	0.024
**Investigations**								
**Thyroid function tests**								
Yes					0.95 (0.37–2.47)	0.920	0.40 (0.11–1.42)	0.155
No^b^					1		1	
**Parental karyotype**								
Yes					0.51 (0.17–1.51)	0.224	0.09 (0.01–0.79)	0.029
No^b^					1		1	

a Reference category: no pregnancy.

b 1 denotes reference category.

* Model 1 included maternal characteristics; Model 2 added the recurrent miscarriage investigations.

Additional analyses were performed to examine whether women who smoked or who had an abnormal karyotype had other characteristics which could account for differences in reproductive outcomes (see Supplementary Table SIII). Smokers were younger (41.7% of those ≤30 smoked compared to 12.6–15.9% of women in the other age categories, *P* < 0.001), with a BMI ≤25 (*P* = 0.043) and 50% of smokers had no previous livebirths compared to 40% of non-smokers (50.6% versus 40.6%, *P* = 0.09). No other characteristics were associated with karyotype.

## Discussion

### Principal findings

This retrospective cohort study aimed to examine the subsequent pregnancy outcomes in women with at least three consecutive miscarriages, and to determine whether any maternal characteristics, investigations or recommended treatments were associated with subsequent pregnancy outcomes.

Women attending the RM clinic were older (64% >35 years) and slightly more likely to experience secondary RM than primary RM. Of the attending women, 74% of women had no medical history; however, notable proportions had a previous gynaecological procedure (19%) or a history of infertility (19%). While maternal age, smoking status and parental karyotype were all associated with pregnancy outcomes, no investigations or treatments were associated with any outcomes.

### Strengths and limitations

This was a large retrospective cohort study of 748 women with consecutive RM. Women included in this study had at least three confirmed consecutive miscarriages; in contrast, previous large register-based studies have not been able to confirm that the secondary RMs were consecutive, or they were restricted to solely examining outcomes after primary RM (Buchmayer *et al.*, 2004; Bhattacharya *et al.*, 2010; Gunnarsdottir *et al.*, 2014; Field and Murphy, 2015; Oliver-Williams *et al.*, 2015). Moreover, this study provides information on investigations and treatments, which is also omitted in larger published cohorts. Few women were lost to follow-up (n = 19, Fig. 1). As women attended a single hospital in their subsequent pregnancy, they were treated consistently and given similar support in follow-up, including bereavement midwife support, early ultrasounds, antenatal care and miscarriage management.

Nonetheless, this work covers a 13-year period, and while the RM clinic staffing and care structures are mainly unchanged, certain aspects of supportive care for RM patients has adapted to reflect up-to-date evidence and greater public awareness and expectations (Meaney *et al.*, 2017; van den Berg *et al.*, 2018). While women from early years in the study had a long follow-up period, this is much shorter for women attending the clinic in later years, potentially underestimating their eventual livebirth rate. Initial data collection was completed by a team of health care professionals with clinical experience in RM. While a data collection protocol was followed, it does not rule out potential discrepancies in data collection. Every effort was made to locate missing data and data were re-checked for accuracy by the primary author. Nonetheless, women may not always present to hospital with a miscarriage, particularly in very early pregnancy, thus the subsequent miscarriage rate is potentially underestimated. As the sole tertiary referral hospital in the region, however, our rate of return is high and, anecdotally, we find women do report early miscarriages to our CMS in Bereavement and Loss and thus the potential omissions should be minimal. Similarly with livebirths, as the largest maternity hospital in the region, transfer of care is uncommon and is usually documented in the chart, with the decision to transfer care often relayed to the Bereavement CMS or evident in the electronic health record. For those women who did not conceive, it was not possible to determine definitively their reproductive intentions, i.e. whether they attempted to conceive again or sought private fertility treatments. Therefore, ‘no further pregnancy’ cannot be assumed to be infertility following RM and should be interpreted with caution. Whether or not women attempted conception, it is important to include women with no further pregnancy in the analysis, to identify factors that may be associated with not conceiving and provides relevant clinical information with which to counsel women. The decision to not pursue further pregnancy after RM is also an important issue that requires greater exploration in qualitative studies.

The introduction of the electronic health record in 2017 significantly improved the availability of some data compared to paper charts. However, data collection in this cohort remains problematic. Women experiencing miscarriage were not formally booked into an antenatal clinic, thus some information was obtained solely from clinical correspondence. BMI was only recorded as ‘normal’ or ‘raised’ in some clinical correspondence, determining how this was reported. Additionally, relevant negative findings such as non-smoking status, BMI <25, no significant medical, surgical or partner histories or investigative findings (particularly pelvic ultrasound) may not have been explicitly stated in clinic letters accounting for levels of missing data for some variables. We are limited to reporting those medications prescribed in the clinic, rather than adherence to prescribed medications. However, electronic antenatal records were checked for the years 2017–2020, which suggested compliance of 90% where information was available (n = 179/198). Similarly, attendance at a consultant-led perinatal medicine clinic was suggested for all women and examination of the electronic records from 2017 to 2020 showed 55% (n = 94/169) attended a perinatal medicine clinic, 26% chose private care (43/169) and 19% received routine antenatal care (32/169).

### Interpretation of results in the context of available literature

Our findings confirm that advanced maternal age, smoking and having a medical or infertility history in addition to experiencing RM is associated with subsequent infertility (Field and Murphy, 2015; Quenby *et al.*, 2021). The majority of women had unexplained RM (60%), consistent with previous studies (Clifford *et al.*, 1994; Jaslow *et al.*, 2010; Fawzy *et al.*, 2016). Only 19% of women had chromosomal analysis performed on products of conception, but foetal aneuploidy was the most common investigative finding. The low rate of chromosomal analysis performed for women attending the clinic may be due to miscarriages at early gestations, complete miscarriage, loss of pregnancy tissue prior to analysis, culture failure or lack of awareness regarding the need for testing upon a third miscarriage, and therefore this merits further scrutiny through clinical audit. The chromosomal analysis demonstrated foetal aneuploidy in 79% of samples, which mirrors the rate of 80% identified in other studies (Marquard *et al.*, 2010). This finding reiterates the relationship between aneuploidy and miscarriage and the significance of chromosomal analysis of products of conception as an explanatory investigation (Foyouzi *et al.*, 2012).

ANA were another frequent positive investigative finding; however, their association with RM is not fully understood to be wholly attributable to recurrent losses (Cavalcante *et al.*, 2020). Overall, 12% of women had a finding of ANA, which is slightly lower than quoted rates in women with RM in a recent systematic review (13.2–50%) (Cavalcante *et al.*, 2020), the rate found in our cohort of women with two consecutive miscarriages (15%), and more in keeping with rates seen in women without RM (0.9–16%) (Green and O’Donoghue, 2019; Cavalcante *et al.*, 2020). This suggests a weaker association between ANA and RM in our cohort, but this is limited by sample size, so it is difficult to draw conclusions. Of the women in our cohort, 8% had abnormal thyroid function tests, which is close to the 7.2% rates previously quoted in RM cohorts (Jaslow *et al.*, 2010). Thrombophilia screening in our cohort found a 5% rate of FVL which was lower than rates in similar cohorts (7.2–17%), and other thrombophilias such as PT gene mutation (2.0%) and APLS (1.4%) were comparatively much lower in our cohort compared to other RM cohorts in a systematic review (Van Dijk *et al.*, 2020), but were in keeping with a more recent cohort study which demonstrated rates of 2.9% and 0.5% for PT gene mutations and APLS, respectively (Shehata *et al.*, 2022). Larger cohort studies are required to determine the clinical value of thrombophilia testing in women with RM.

Parental karyotyping demonstrated balanced translocations in 4% of parents, which is comparable to the wider literature (Van Dijk *et al.*, 2020). Parental karyotype was the only investigation shown to be significantly associated with pregnancy outcome; these parents were more likely not to conceive than have a miscarriage or a livebirth. We suggest that this likely represents the additional considerations for these parents to embark on a subsequent pregnancy, such as the potential for deferral in conception to consult a clinical geneticist or consideration of ART to facilitate pre-implantation genetic testing, although conception rates and livebirths in parents with balanced translocations are not significantly reduced among those who choose to conceive naturally over ART (Kabessa *et al*., 2018; Li *et al*., 2021). Furthermore, these couples were equally likely to be nulliparous and were not significantly older than those with a normal karyotype.

Most women received high-dose folic acid and low-dose aspirin with supportive care for a future pregnancy. Folic acid is prescribed to reduce the risks of neural tube defects, particularly in women with obesity, epilepsy or diabetes, particularly in the Irish population as there is no fortification of food (Turner, 2018; Egan *et al*., 2021). However, folic acid has not been shown to reduce the risk of miscarriage in women with RM (ESHRE Early Pregnancy Guideline Development Group, 2017). Aspirin was prescribed to almost all women attending the RM clinic. Rather than reducing subsequent miscarriage risk, aspirin was given to reduce placental dysfunction in a subsequent pregnancy, to reduce the risk of preeclampsia and intra-uterine growth restriction, particularly for women aged >35 years, smokers, women with hypertension or women undergoing ART (Van Oppenraaij *et al.*, 2009; Gunnarsdottir *et al.*, 2014; Bartsch *et al.*, 2016). The rate of LMWH and aspirin prescription is much higher than the rate of thrombophilia within the cohort, which is in keeping with the findings of Manning *et al.* (2020). This combination has not been shown to be of benefit to women with RM without APLS (Coomarasamy *et al.*, 2021). At a local level, these prescribing practices merit greater scrutiny, but should be examined alongside other relevant pregnancy outcomes such as pre-eclampsia or foetal growth restriction to assess any attributable benefits and in distinct cohorts. For example, women attending with two miscarriages and prescribed any medication were more likely to have a livebirth than those given no medication (Green and O’Donoghue, 2019).

Progesterone was prescribed to 56% of women with RM. An updated meta-analysis suggests progesterone may reduce miscarriage in RM, particularly in first-trimester bleeding or with higher-order miscarriage and should be considered (Coomarasamy *et al.*, 2021). Our unadjusted regression model suggested that women prescribed progesterone were more likely to have a miscarriage than no further pregnancy. Our interpretation is that this represents how this group are more likely to attempt conception following attendance at the RM clinic than not, which is mirrored in the high rate of conception in the first 12 months after attending the RM clinic (77%), as seen previously (Kaandorp *et al.*, 2014). In the adjusted model, however, this association was not significant. Prednisolone, hydroxychloroquine and metformin were prescribed infrequently, in keeping with international guidance (ESHRE Early Pregnancy Guideline Development Group, 2017). No other prescribed treatments had a significant association with outcome in our cohort, echoing findings in the international literature (Coomarasamy *et al.*, 2021).

The livebirth rates of 48% overall and 63% in women with subsequent pregnancies is lower than rates reported in the literature of 74–86% (Fawzy *et al.*, 2008; Dempsey *et al.*, 2015; Ali *et al.*, 2020; Ticconi *et al.*, 2020). It is also lower than the livebirth rate of 73% reported in women attending our RM clinic with two miscarriages (Green and O’Donoghue, 2019). Almost a quarter of women had no further pregnancies; this may be due to the shorter follow-up period for a minority (52/742 (7%) were followed for <24 months). When including successive pregnancies, the overall livebirth rate climbed from 48% to 63%, but remained lower for women with infertility issues (54%) and those over 40 (44%). Notably, over a quarter of our cohort was aged over 40 years and 67% in this age group had a living child. These factors, which may influence whether couples pursue a further pregnancy or not, merit further exploration. The psychological impact of RM itself may also influence this decision (Shields *et al.*, 2022). Qualitative studies are required to better understand patient experiences, particularly those of women/couples with infertility (Schwerdtfeger and Shreffler, 2009). Women aged 30–34 and 35–39 years were more likely to have a livebirth than no further pregnancy, and women aged 30–34 years were also more likely to have a miscarriage than no further pregnancy. This reflects greater fecundity in this age group and desire to conceive, as mentioned previously. Smoking is well recognized as a risk factor for infertility and miscarriage (Quenby *et al.*, 2021) and smokers in our cohort were significantly less likely to have a subsequent pregnancy despite being younger and having no prior livebirths.

It is notable that there was little information available on male partner history and so this data was excluded from the analysis. This is reflective of long-standing bias within research on the developmental origins of health and disease that maternal pregnancy effects are of greater influence than paternal contributions (Sharp *et al.*, 2018, 2019). The role of male health, in particular sperm quality, in RM is increasingly recognized (Schlegel *et al.*, 2021). The psychological impact of RM on male partners is well established, but may not be acknowledged in the hospital setting, leaving male partners feeling unsupported and unimportant (Williams *et al.*, 2020; Harty *et al.*, 2022). Healthcare professionals must take a holistic and couple-focused approach to RM, to better meet their psychological needs (Koert *et al.*, 2019).

### Clinical implications

These findings provide useful information with which to counsel women or couples with RM. The findings are largely reassuring with the majority of women conceiving over time. For those women who smoke, have a balanced translocation, are older or have a concurrent fertility issue, our findings can alert the women and their clinicians to their potential reduced likelihood of conception and livebirth and thus allow for greater counselling regarding individual risk factors as well as facilitating additional supports.

### Research implications

This research demonstrates important information on the reproductive outcomes following RM. There is a need for larger similar studies with greater details on pregnancy outcomes. While antenatal and delivery complications were beyond the scope of this current paper, it is recognized that preeclampsia, gestational diabetes, preterm birth, placental abruption and stillbirth are associations with RM (Gunnarsdottir *et al.*, 2014; Field and Murphy, 2015; Ticconi *et al.*, 2020; Ausbeck *et al.*, 2021; Wu *et al.*, 2022). Induction of labour and caesarean section rates in subsequent pregnancies have also been reported as increased among women with RM (Field and Murphy, 2015). The lack of a national register on miscarriage remains a significant limitation in obtaining accurate data on miscarriage and subsequent pregnancy outcomes. Alternatively, consideration should be given to a local prospective database, such as recently employed by ‘Tommy’s Net’ to better capture this data (Shields *et al.*, 2022). Qualitative work is also needed to explore couples’ experiences of trying to conceive after pregnancy loss, including cessation of trying, and to identify the supports and needs of these couples.

## Conclusion

Following RM, 77% of women had a subsequent pregnancy, of whom 63% of women had a livebirth. Younger women were more likely to conceive than to have no further pregnancy. Women over 40 or with infertility were also less likely to have a livebirth over time. In addition to older age, smoking and parental balanced translocations were associated with a reduced likelihood of further pregnancy. No investigation or treatment was associated with pregnancy outcome, reiterating the importance of the supportive aspects of care for women and their partners after RM, as well as counselling regarding individual risk factors. There is a need for greater information on pregnancy outcomes in women with RM, and to facilitate this, consideration should be given to prospective RM databases and a national register.

## Supplementary data

Supplementary data are available at *Human Reproduction Open* online.

## Supplementary Material

hoac045_Supplementary_Data

## Data Availability

The data underlying this article cannot be shared publicly due to the confidential nature of patient information. Data may be made available on reasonable request.
