# Supplementary material for: Reproductive outcomes following recurrent first-trimester miscarriage: a retrospective cohort study
Source: Hum Reprod Open. 2022 Oct 11;2022(4):hoac045. doi: 10.1093/hropen/hoac045 (PMC9632453; doi:10.1093/hropen/hoac045)
Supplement: hoac045_Supplementary_Data [file hoac045_supplementary_data.docx]

## **Supplementary Table SI. Maternal Characteristics versus Pregnancy Outcome**

| Maternal Characteristic (n, %) | Livebirth n (%)  (N=359) | Pregnancy Loss (N=208) | p-value |
| --- | --- | --- | --- |
| Maternal age  ≤29  30-34  35-39  ≥40 | 45 (68.2)  102 (65.4)  151 (69.3)  61 (48.0) | 21 (31.8)  54 (34.6)  67 (30.7)  66 (52.0) | <0.001 |
| Previous Livebirth  Yes  No | 202 (64.5)  157 (61.8) | 111 (35.5)  97 (38.2) | 0.953 |
| Medical history  Yes  No | 95 (61.3)  259 (64.3) | 60 (38.7)  144 (35.7) | 0.513 |
| Smoker  Yes  No | 35 (63.6)  212 (64) | 20 (36.4)  119 (36) | 0.953 |
| Fertility History  Yes  No | 58 (59.2)  301 (64.2) | 40 (40.8)  168 (35.8) | 0.351 |
| Thyroid function tests  Normal  Abnormal | 329 (62.7)  28 (71.8) | 196 (37.3)  11 (28.2) | 0.254 |
| Parental Karyotype  Balanced translocation present  Normal karyotype | 13 (81.2)  326 (62.8) | 3 (18.8)  193 (37.2) | 0.132 |

## **Supplementary Table SII. Unadjusted Multinomial Regression Analysis Maternal Characteristics versus Reproductive Outcome**

| Variable | Reproductive Outcome | | | | |
| --- | --- | --- | --- | --- | --- |
|  | **Live birth ^a^ (n =359)** | | **Pregnancy Loss^a^ (n=208)** | |  |
| Age Category | **RRR (95% CI) p-value** | | **RRR (95% CI) p-value** | |  |
| <29  30-34  35-39  >40 | 2.213 (1.19-4.103)  4.598 (2.614-8.088)  2.593 (1.644-4.09)  1^b^ | 0.012  <0.01  <0.01 | 0.955 (0.48-1.9)  2.25 (1.248-4057)  1.063 (0.655-1.726) | 0.895  0.007  0.803 |  |
| Previous Livebirth  Yes  No | 1  1.012 (0.703-1.458) | 0.947 | 1  1.138(0.76-1.706) | 0.53 |  |
| Medical history  Yes  No | 0.654 (0.441-0.97)  1 | 0.035 | 0.743 (0.48-1.15)  1 | 0.182 |  |
| Gynaecological condition  Yes  No | 0.517 (0.257-1.041)  1 | 0.065 | 0.712 (0.337-1.505)  1 | 0.374 |  |
| Gynaecological Procedure  Yes  No | 1  0.714 (0.457-1.115) | 0.139 | 1  0.594(0.418-1.151) | 0.157 |  |
| BMI  <25  >25 | 1.1 (0.549-2.205)  1 | 0.787 | 1.038 (0.479-2.25) | 0.924 |  |
| Smoker  Yes  No | 0.461 (0.265-0.803)  1 | 0.006 | 0.469(0.249-0.887)  1 | 0.02 |  |
| Fertility History  Yes  No | 0.61 (0.39-0.954)  1 | 0.03 | 0.754 (0.462-123)  1 | 0.258 |  |
| ART History  Yes  No | 1.178 (0.6-2.312)  1 | 0.634 | 1.474 (0.719-3.02)  1 | 0.289 |  |
| Factor V Leiden  Mutation Present  Mutation Absent | 1.081 (0.482-2.427)  1 | 0.85 | 0.546 (0.19-1.566)  1 | 0.26 |  |
| Anti-Cardiolipin Antibodies  Present  Absent | 2.94 (0.351-24.614)  1 | 0.32 | 3.44 (0.381-31.070)  1 | 0.271 |  |
| All autoantibodies  1 or more present  No antibodies | 1.066 (0.621-1.831)  1 | 0.816 | 0.9 (0.486-1.669)  1 | 0.738 |  |
| HbA1c  Elevated  Not elevated | 0.319 (0.053-1.928)  1 | 0.213 | 0.553 (0.091-3.347)  1 | 0.519 |  |
| Thyroid function tests  Abnormal  Normal | 0.685 (0.371-1.265)  1 | 0.227 | 0.452 (0.209-0.978)  1 | 0.044 |  |
| Previous Fetal karyotype  Aneuploid  Euploid | 2.652 (0.957-7.344)  1 | 0.061 | 1.1 (0.403-3.002)  1 | 0.852 |  |
| Parental Karyotype  Balanced translocation present  Normal karyotype | 0.479 (0.213-1.074)  1 | 0.074 | 0.187 (0.052-0.673)  1 | 0.01 |  |
| Pelvic US  Finding on US  No finding | 1.146 (0.514-2.557)  1 | 0.739 | 1.434 (0.611-3.361)  1 | 0.407 |  |
| Any positive investigation finding  Yes  No | 0.945 (0.657-1.36)  1 | 0.76 | 0.859 (0.572-1.29)  1 | 0.465 |  |
| Prescribed Aspirin  Yes  No | 1.063 (0.421-2.686)  1 | 0.897 | 0.965 (0.352-2.648)  1 | 0.945 |  |
| Prescribed Folic Acid 5mg  Yes  No | 0.866 (0.565-1.327)  1 | 0.508 | 1.171 (0.719-1.907)  1 | 0.526 |  |
| Prescribed Progesterone  Yes  No | 1.013 (0.699-1.468)  1 | 0.945 | 1.717 (1.132-2.603)  1 | 0.011 |  |
| Prescribed LMWH  Yes  No | 0.775 (0.504-1.192)  1 | 0.246 | 1.046 (0.654-1.671)  1 | 0.852 |  |
| Prescribed Prednisolone  Yes  No | 1.416 (0.45-4.459)  1 | 0.552 | 2.268 (0.709-7.259)  1 | 0.168 |  |
| Prescribed Metformin  Yes  No | 0.576 (0.153-2.175)  1 | 0.416 | 0.594 (0.131-2.693)  1 | 0.499 |  |
| ^a^Reference category: no pregnancy; ^b^1 denotes reference category | | | | | |

| Maternal Characteristic (n, %) | Smoker | Non-smoker | p-value |
| --- | --- | --- | --- |
| Maternal age  ≤29  30-34  35-39  ≥40 | 25 (41.7)  20 (15.9)  25 (12.6)  15 (12.9) | 35 (58.3)  106 (84.1)  174 (87.4)  101 (87.1) | <0.001 |
| Previous Livebirth  Yes  No | 42 (49.4)  43 (50.6) | 247 (59.4)  169 (40.6) | 0.09 |
| Medical history  Yes  No | 29 (35.4)  53 (64.6) | 290 (70.6)  121 (29.4) | 0.177 |
| Gynae history  Yes  No | 5 (6.1)  77 (93.9) | 34 (8.2)  380 (91.8) | 0.287 |
| Fertility History  Yes  No | 12(14.1)  73 (85.9) | 93 (22.4)  323 (77.6) | 0.089 |
| BMI  ≤25  >25 | 9 (37.5)  15 (62.5) | 49 (19.8)  199 (80.2) | 0.043 |
| Maternal Characteristic (n, %) | **Balanced translocation present** | **Normal karyotype** | **p-value** |
| Maternal age  ≤29  30-34  35-39  ≥40 | 2 (2.4)  5 (2.8)  3 (16)  5 (2.8) | 83 (97.6)  171 (97.2)  241 (93.8)  174 (97.2) | 0.159 |
| Previous Livebirth  Yes  No | 15 (53.6)  13 (46.4) | 308 (46.0)  361 (54.0) | 0.434 |
| Medical history  Yes  No | 7 (25.9)  20 (74.1) | 202 (30.9)  452 (69.1) | 0.584 |
| Gynaecological history  Yes  No | 2 (7.1)  26 (92.9) | 44 (6.8)  605 (93.2) | 0.940 |
| Fertility History  Yes  No | 7 (25)  21 (75) | 126 (18.8)  543 (81.2) | 0.416 |
| BMI  ≤25  >25 | 2 (28.6)  5 (71.4) | 63 (24.4)  195 (75.6) | 0.801 |
| Smoker  Yes  No | 1 (5.6)  17 (94.4) | 81 (17.8)  373 (82.8) | 0.177 |

## **Supplementary Table SIII. Maternal Characteristics according to Smoking and Parental Karyotype**
